# Supplementary material for: Modeling glioblastoma heterogeneity as a dynamic network of cell states
Source: Mol Syst Biol. 2021 Sep 16;17(9):e10105. doi: 10.15252/msb.202010105 (PMC8444284; doi:10.15252/msb.202010105)
Supplement: Supplementary file 6 — Source Data for Figure 5 [file MSB-17-e10105-s004.zip › Figure5A_sourcedata/GSEA_3071/nosigdb_stateV.GseaPreranked.1622795159954/WANG_MESENCHYMAL_PMID_28697342.html]

Details for gene set WANG\_MESENCHYMAL\_PMID\_28697342[GSEA]

|  || Dataset | state43071 |
| Phenotype | NoPhenotypeAvailable |
| Upregulated in class | na\_neg |
| GeneSet | WANG\_MESENCHYMAL\_PMID\_28697342 |
| Enrichment Score (ES) | -0.8331783 |
| Normalized Enrichment Score (NES) | -3.519196 |
| Nominal p-value | 0.0 |
| FDR q-value | 0.0 |
| FWER p-Value | 0.0 |
Table: GSEA Results Summary

  

Fig 1: Enrichment plot: WANG\_MESENCHYMAL\_PMID\_28697342      
 Profile of the Running ES Score & Positions of GeneSet Members on the Rank Ordered List

  

| PROBE | GENE SYMBOL | GENE\_TITLE | RANK IN GENE LIST | RANK METRIC SCORE | RUNNING ES | CORE ENRICHMENT || 1 | COL1A2 |  |  | 1028 | 0.614 | -0.1786 | No |
| 2 | IL7R |  |  | 1761 | 0.425 | -0.3060 | No |
| 3 | SDC1 |  |  | 2491 | 0.323 | -0.4357 | No |
| 4 | SYNGR2 |  |  | 2933 | 0.276 | -0.5119 | No |
| 5 | GPRC5A |  |  | 3483 | -0.297 | -0.6081 | No |
| 6 | ITGB1 |  |  | 3614 | -0.317 | -0.6242 | No |
| 7 | VDR |  |  | 3793 | -0.348 | -0.6485 | No |
| 8 | LY96 |  |  | 3829 | -0.354 | -0.6456 | No |
| 9 | ARPC1B |  |  | 4818 | -0.730 | -0.8134 | Yes |
| 10 | BNC2 |  |  | 4819 | -0.730 | -0.7936 | Yes |
| 11 | CNN2 |  |  | 4840 | -0.749 | -0.7771 | Yes |
| 12 | NNMT |  |  | 5004 | -0.942 | -0.7825 | Yes |
| 13 | CTSB |  |  | 5007 | -0.948 | -0.7572 | Yes |
| 14 | TNFAIP8 |  |  | 5009 | -0.949 | -0.7317 | Yes |
| 15 | FTL |  |  | 5072 | -1.077 | -0.7143 | Yes |
| 16 | LOX |  |  | 5092 | -1.128 | -0.6874 | Yes |
| 17 | GLIPR1 |  |  | 5109 | -1.177 | -0.6585 | Yes |
| 18 | TGFBI |  |  | 5136 | -1.259 | -0.6293 | Yes |
| 19 | P4HA2 |  |  | 5164 | -1.393 | -0.5967 | Yes |
| 20 | PLAUR |  |  | 5185 | -1.479 | -0.5605 | Yes |
| 21 | PRSS23 |  |  | 5189 | -1.489 | -0.5207 | Yes |
| 22 | COL15A1 |  |  | 5190 | -1.490 | -0.4803 | Yes |
| 23 | S100A11 |  |  | 5196 | -1.536 | -0.4397 | Yes |
| 24 | THBS1 |  |  | 5197 | -1.538 | -0.3980 | Yes |
| 25 | COL5A1 |  |  | 5274 | -2.455 | -0.3459 | Yes |
| 26 | LUM |  |  | 5277 | -2.507 | -0.2783 | Yes |
| 27 | CAV1 |  |  | 5279 | -2.605 | -0.2080 | Yes |
| 28 | COL3A1 |  |  | 5292 | -3.630 | -0.1119 | Yes |
| 29 | COL1A1 |  |  | 5294 | -4.144 | 0.0002 | Yes |
Table: GSEA details [plain text format]

  

Fig 2: WANG\_MESENCHYMAL\_PMID\_28697342: Random ES distribution      
 Gene set null distribution of ES for **WANG\_MESENCHYMAL\_PMID\_28697342**
